# Functional impairment of Tax-specific but not CMV-specific CTLs in a minor population of asymptomatic HTLV-1-carriers

**DOI:** 10.1186/1742-4690-8-S1-A101

**Published:** 2011-06-06

**Authors:** Atsuhiko Hasegawa, Ayako Takamori, Atae Utsunomiya, Yasuhiro Maeda, Yoshihisa Yamano, Yukiko Shimizu, Yotaro Tamai, Amane Sasada, Na Zeng, Ilseung Choi, Naokuni Uike, Jun Okamura, Toshiki Watanabe, Takao Masuda, Mari Kannagi

**Affiliations:** 1 Department of Immunotherapeutics, Tokyo Medical and Dental University, Tokyo, 113-8519, Japan; 2 Department of Hematology, Imamura Bun-in Hospital, Kagoshima, Japan; 3 Division of Hematology, Department of Internal Medicine, Kinki University School of Medicine, Osaka, Japan; 4 Department of Molecular Medical Science, Institute of Medical Science, St. Marianna University School of Medicine, Kawasaki, Japan; 5 Department of Hematology, National Kyushu Cancer Center, Fukuoka, Japan; 6 Institute for Clinical Research, National Kyushu Cancer Center, Fukuoka, Japan; 7 Laboratory of Tumor Cell Biology, Department of Medical Genome Science, Graduate School of Frontier Sciences, The University of Tokyo, Tokyo, Japan

## 

Human T-cell leukemia virus type 1 (HTLV-1) causes adult T-cell leukemia (ATL) and HTLV-I-associated myelopathy/tropical spastic paraparesis (HAM/TSP) in a small percentage of infected individuals. ATL is often associated with general immune suppression and an impaired HTLV-1-specific T cell response. We previously found that a small fraction of asymptomatic HTLV-1-carriers (AC) also showed impaired T cell responses against the major target antigen Tax. However, it is unclear whether the impaired HTLV-1-specific T cell response in these individuals is an HTLV-1-specific phenomenon, or merely reflects general immune suppression. In this study, we investigated the function of Tax-specific cytotoxic T lymphocytes (CTLs) using tetramers consisting of major CTL epitopes and HLA-A0201, A2402, or A1101 in various HTLV-1-infected individuals possessing these HLAs. Tax-specific CTLs were detected in 33.3% (n=7/21), 100% (n=18/18), and 86.4% (n=19/22) of chronic ATL (cATL) patients, HAM/TSP patients, and AC, respectively. Tax-specific CTLs of all HAM/TSP patients tested proliferated well in culture and produced IFN-γ when stimulated with Tax peptides, while Tax-specific CTLs detected in cATL patients did not. In ACs, the Tax-specific CTL responses were retained in most cases. However, Tax-specific CTLs in one AC hardly produced IFN-γ and failed to proliferate or express a degranulation (CD107a) marker upon Tax peptide stimulation. In contrast, cytomegalovirus (CMV) pp65-specific CTLs in the same donor could be fully activated by pp65 peptide stimulation. These findings indicated that HTLV-1-specific T cell function is impaired in a limited population in an HTLV-1-specific manner during an asymptomatic stage.

